# Introgression and monitoring of wild *Helianthus praecox* alien segments associated with Sclerotinia basal stalk rot resistance in sunflower using genotyping-by-sequencing

**DOI:** 10.1371/journal.pone.0213065

**Published:** 2019-03-01

**Authors:** Zahirul I. Talukder, Yunming Long, Gerald J. Seiler, William Underwood, Lili Qi

**Affiliations:** 1 Department of Plant Sciences, North Dakota State University, Fargo, North Dakota, United States of America; 2 Sunflower and Plant Biology Research Unit, USDA-Agricultural Research Service, Edward T. Schafer Agricultural Research Center, Fargo, North Dakota, United States of America; HudsonAlpha Institute for Biotechnology, UNITED STATES

## Abstract

Sclerotinia basal stalk rot (BSR) and downy mildew are major diseases of sunflowers worldwide. Breeding for BSR resistance traditionally relies upon cultivated sunflower germplasm that has only partial resistance thus lacking an effective resistance against the pathogen. In this study, we report the transfer of BSR resistance from sunflower wild species, *Helianthus praecox*, into cultivated sunflower and molecular assessment of the introgressed segments potentially associated with BSR resistance using the genotyping-by-sequencing (GBS) approach. Eight highly BSR-resistant *H*. *praecox* introgression lines (ILs), H.pra 1 to H.pra 8, were developed. The mean BSR disease incidence (DI) for H.pra 1 to H.pra 8 across environments for four years ranged from 1.2 to 11.1%, while DI of Cargill 270 (susceptible check), HA 89 (recurrent parent), HA 441 and Croplan 305 (resistant checks) was 36.1, 31.0, 19.5, and 11.6%, respectively. Molecular assessment using GBS detected the presence of *H*. *praecox* chromosome segments in chromosomes 1, 8, 10, 11, and 14 of the ILs. Both shared and unique polymorphic SNP loci were detected throughout the entire genomes of the ILs, suggesting the successful transfer of common and novel introgression regions that are potentially associated with BSR resistance. Downy mildew (DM) disease screening and molecular tests revealed that a DM resistance gene, *Pl*_*17*_, derived from one of the inbred parent HA 458 was present in four ILs. Introgression germplasms possessing resistance to both Sclerotinia BSR and DM will extend the useful diversity of the primary gene pool in the fight against two destructive sunflower diseases.

## Introduction

Cultivated sunflower (*Helianthus annuus* L.) is an important oilseed and confection crop worldwide. Fungal diseases caused by *Sclerotinia sclerotiorum* are of concern in sunflower production in the United States, as well as other parts of the world causing millions of dollars of crop losses annually [1]. *S*. *sclerotiorum* causes infection of sunflower plants at any growth stage and results in so-called Sclerotinia wilt or basal stalk rot (BSR), mid-stalk rot (MSR), and head rot (HR) diseases [2]. Sclerotinia BSR and HR are the most common sunflower diseases in the humid temperate production areas of Argentina, Europe, China, and the Northern Great Plains, where most of the U.S. sunflower crop is grown. The mode of infection for the two diseases differs. BSR is initiated by root infection from mycelia (unique to sunflower), while HR is caused by germination of airborne ascospores on sunflower capitula. Despite the common causal agent, the inheritance of resistance to Sclerotinia BSR and HR in sunflower is different based on the lack of a relationship between the two forms of the diseases [3,4]. Therefore, specialized screening nurseries and inoculation procedures are required for breeding of the two forms of Sclerotinia resistance, which effectively doubles the effort to combat the loss caused by the fungus.

BSR is the predominant Sclerotinia disease of sunflower in the Northern Great Plains [3]. The sclerotia produced by the fungus plays a major role in the BSR disease cycle in the field. Under favorable environmental conditions, the mycelia from germinating sclerotia incite sunflower root infection. Typical BSR symptoms are revealed as a light-brown lesion girdling the stalk at soil level and extend up a few inches with the occasional presence of white, cottony mycelial growth visible under favorable moisture conditions. Over time, the fungus grows internally, destroying the pith and causing the plant to wilt and gradually die [2]. Chemical control of BSR is not feasible because the infection begins below ground. There is no effective fungicide registered in the U.S. and other countries for controlling BSR in sunflower. Therefore, breeding of resistant hybrids is the most efficient, economical, and environmentally friendly disease management approach.

Breeding for BSR resistance is challenging, since no immune germplasm has thus far been identified in sunflower or its close relatives. Nevertheless, various studies have demonstrated that resistance performance of diverse sunflower germplasms differs considerably [4,5,6] and the resistance is conditioned by multiple genes, each having a small effect [7,8,9]. Davar et al. [7] identified seven QTL associated with BSR resistance on LGs 1, 2, 4, 6, 8, 14 and 17 in a sunflower recombinant inbred line (RIL) mapping population of 116 individuals derived from the cross of PAC2/RHA266. Amouzadeh et al. [8] screened 99 RILs of the same population, but with a different fungal isolate and identified five QTL on LGs 1, 3, 8, 10 and 17. The effects of QTL for both studies were small (0.5–8%) with the QTL intervals ranging from 1.8 to 18.6 cM. Talukder et al. [9] identified two BSR resistance QTL on LG10 and LG17 using integrated data from multiple environments explaining 32 and 15%, respectively of the observed phenotypic variance. An additional four environment specific QTL were also identified on LGs 4, 9, 11 and 16, each explaining between 6 and 10% of the phenotypic variances. An association mapping effort identified two candidate genes on LG14 of the sunflower genome associated with BSR resistance [10]. Sclerotinia resistance in sunflower has traditionally been accomplished using classical genetic research and breeding efforts, often utilizing the available genetic variation in the primary gene-pool. However, the genetic base of the cultivated sunflower is narrow. Co-ancestry analysis has revealed that public sunflower inbred lines have originated from a small number of ancestral germplasm sources [11]. The consequence of the so-called founder effect, as defined by Ladizinsky [12], makes sunflower vulnerable to many biotic and abiotic stresses. Resistance present in the currently available cultivated sunflower gene-pool is not sufficient against the threat posed by the Sclerotinia [4,9,13]. Therefore, there is a growing need to improve Sclerotinia resistance in cultivated sunflower by diversifying its genetic variability utilizing the sunflower crop wild relatives. An abundance of sunflower crop wild relatives occupying a variety of habitats in the continental USA where they coevolved in the center of origin, are a valuable resource in the fight against biotic and abiotic stresses [14].

Cultivated sunflower belongs to the genus *Helianthus*, a member of the Asteraceae family consisting of 53 species, including 14 annual and 39 perennial [14]. All annual wild *Helianthus* species are diploid (2n = 2x = 34) and readily crossable with cultivated sunflower (except *H*. *agrestis*) with limited incompatibility, and homoeologous recombination occurs with relative ease. Genetic resistance has been identified in wild *Helianthus* species for sunflower rust (caused by *Puccinia helianthi* Schwein.), and downy mildew (caused by *Plasmopara halstedii* (Farl.) Berl. Et de Toni) and are routinely being deployed into cultivated sunflower as race-specific single dominant genes [15–20]. Earlier studies have repeatedly demonstrated high level of Sclerotinia resistance in the wild *Helianthus* gene-pool (reviewed by Seiler et al. [14]). Despite the devastating impact on the sunflower, it is apparent that wild *Helianthus* resources have not been adequately utilized for Sclerotinia resistance breeding. This limitation was partly due to the complex quantitative nature of the BSR resistance and the unavailability of efficient genomic tools to simultaneously assess multiple introgression regions in the cultivated sunflower background. However, the recent release of the sunflower reference genome sequence offers new opportunities for sunflower improvement by identifying genes of agronomic interest [21]. The use of high-throughput next-generation sequence (NGS) based genotyping-by-sequencing (GBS) technology in hybridization and introgression studies has increased the potential to identify single nucleotide polymorphism (SNP) variation in specific DNA targets across the entire genome for dissecting complex quantitative traits [22].

*H*. *praecox* Engelm. & A. Gray is an annual wild sunflower species, also known by the common name Texas sunflower. *H*. *praecox* has three subspecies: *H*. *praecox* subsp. *praecox*, *H*. *praecox* subsp. *runyonii* and *H*. *praecox* subsp. *hirtus* [23,24]. All three subspecies are endemic to the state of Texas in the USA, and grow on sandy soils of the coastal prairies. *H*. *praecox* and its hybrid progenies showed a high level of Sclerotinia resistance in various studies [25–31], making the species a valuable source for Sclerotinia resistance genes for introgressing into a cultivated sunflower background.

In the present study, we report the transfer of Sclerotinia BSR resistance from *H*. *praecox* into cultivated sunflower, as well as monitoring alien segments in the highly BSR resistant introgression lines (ILs) using GBS-derived SNP markers. Additionally, we report the integration of a broad-spectrum downy mildew (DM) resistance gene, *Pl*_17_, into BSR resistant ILs derived from one of the parents, HA 458. The germplasms developed and information generated in this study will help breeders expedite resistance breeding against two important sunflower diseases.

## Materials and methods

### Plant materials

Five accessions of *H*. *praecox* (PI 413176, PI 435849, PI 468853, PI 435855, and PI 468847) were selected as BSR resistant donor parents identified by Block et al. [27,28]. These accessions were all collected from Texas, USA. Among the accessions, PI 413176 is subsp. *praecox*, PI 435849 and PI 468853 are subsp. *runyonii*, and PI 435855 and PI 468847 are subsp. *hirtus*. Three inbreed lines HA 89 (PI 599773), nuclear male sterile (NMS) HA 89 (PI 559477), and HA 458 (PI 655009) were used as cultivated sunflower sources. All these lines possess good agronomic traits, but they are susceptible to BSR disease. HA 89 was released in 1971 as an oilseed maintainer line by USDA-ARS and the Texas Agricultural Experiment Station. NMS HA 89 is a mutant developed by streptomycin treatment of HA 89 possessing a recessive gene, *ms9* that controls male sterility [32]. It was released as nuclear male-sterile genetic stock in 1990 [33]. HA 458 was released in 2010 as a high oleic maintainer line carrying the DM resistant *Pl*_*17*_ gene [34,35]. Two commercial sunflower hybrids, Croplan 305 and Cargill 270, were used as resistant and susceptible checks, respectively. Additionally, inbred line HA 441 was also used as a resistant control in each BSR screening test.

### Crossing, backcrossing and generation advance

The selected five wild *H*. *praecox* accessions and NMS HA 89 were grown in the greenhouse. The first round of crosses were made in 2009 with *H*. *praecox* accessions as the male parent and NMS HA 89 as the female parent. A total of 2,131, 1,602, 1,679, 1,383 and 1,721 florets of NMS HA 89 were separately pollinated with pollen from *H*. *praecox* accessions, PI 413176, PI 435849, PI 468853, PI 468847 and PI 435855, respectively, to obtain F_1_ seeds.

Basal stalk rot resistant F_1_ plants were crossed with HA 458. The progenies from these crosses were termed BC_1_s. HA 89 was used as the recurrent female parent to backcross to the selected resistant BC_1_s. The BC_2_F_1_ progenies were selfed and advanced to the BC_2_F_2_ generation, followed by repeated selfing for four generations. The F_1_ through BC_2_F_2_ generations were screened for BSR resistance in the greenhouse, and only resistant progenies were advanced to the next generation. The BC_2_F_3_ families and progenies of the following generations were evaluated for BSR resistance in the field nurseries during 2012 to 2015 with resistant progenies advanced to the next generation.

### BSR screening in the greenhouse

**Fig 1:**
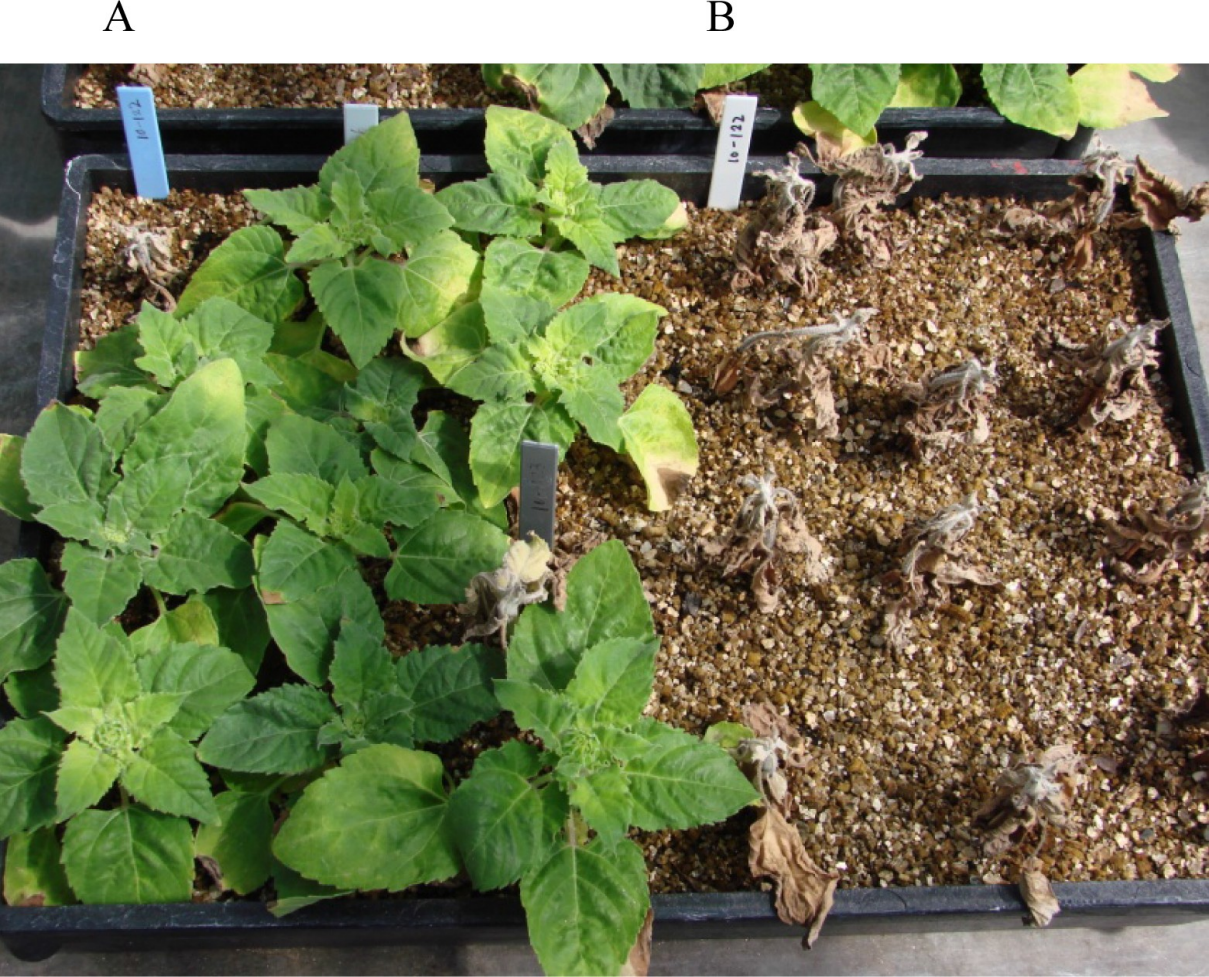
Sclerotinia basal stalk rot evaluations of the F_1_ hybrids in the greenhouse. (A) The F_1_ hybrids of NMS HA 89/*H*. *praecox* PI 468853 on the left half of the tray scored 18 days after inoculation, and dead plants on the left half are susceptible checks with blue and gray labels, respectively, (B) Susceptible check Cargill 270.

The *S*. *sclerotiorum* fungal isolate NEB-274 was used for inoculum production of all greenhouse and field screening trials, as described by Qi et al. [36]. The seeds of each generation (F_1_ to BC_2_F_2_) along with the recurrent parent HA 89, and checks Cargill 270, HA 441, and the Croplan 305, were grown in the greenhouse in plastic flats each containing six rows of four 5.7 × 7.6 cm wells filled with Sunshine SB 100B potting compost (SunGro Horticulture, Bellevue, WA). The inoculation trays (54.6 × 34.3 × 10.2 cm) were prepared by spreading 120 g of inoculums on a layer of vermiculite placed on top of a fiberglass screen at the bottom of each tray. The inoculation trays were then placed in a dark and humid phytotron at ~22°C for three days before they were moved to the greenhouse. Three-week-old sunflower seedlings were carefully uprooted from the plastic flats and placed directly on the inoculums bed of the inoculation trays. The gaps at the base of the seedlings were filled with vermiculite to hold sufficient moisture when watered. The trays were incubated in the greenhouse at a soil temperature of 22–24°C. The inoculated seedlings were visually inspected daily for disease symptoms and were scored at 14–18 days after inoculation (Fig 1). Sclerotinia BSR disease incidence (DI) is expressed as the percentage of dead and/or wilted plants.

### Field experiments

The progenies of BC_2_F_3_ through BC_2_F_5_ families were grown and tested for BSR resistance in the field at Carrington, ND (47.4497° N, 99.1262° W), Grandin, ND (47.2369° N, 97.0015° W), and Crookston, MN (47.7742° N, 96.6078° W) during 2012–2015. In all field trials, the hybrid sunflower Cargill 270 and Croplan 305 were used as the susceptible and resistant checks, respectively. Additionally, an USDA-ARS released inbred line HA 441 was also used as a resistant check. The seeds of the progeny lines, the recurrent parent, and the checks, were planted in 6-m long single row plots with 75-cm row spacing. In each plot, 25 seeds were sown per row with 20 plants kept after emergence for BSR evaluation. The field trials were laid out with a randomized complete block design with two replications in 2012 and 2013 and three replications in 2014 and 2015 per year and location for each ILs. Each field trial was artificially inoculated following the method developed by Gulya et al. [37]. Approximately 90 g of *S*. *sclerotinia* inoculum were applied for each entry in row-side furrows 5–6 weeks after planting at approximately the V6 growth stage [38]. Disease incidence (DI) was used as an index of BSR susceptibility measured at physiological maturity of the sunflower plants in the field at the R9 growth stage [38]. DI was calculated as the percentage of plants showing BSR symptoms in each row.

### Statistical analysis

Analysis of variance (ANOVA) was performed for the replicated field tests data using the PROC Mixed of SAS version 9.4 [39]. Each location of individual year was considered as an environment. The genotypes were considered as fixed, while the remaining factors were treated as random effects using the model:

$$Y_{ijk}=\mu +l_{i}+b(l{)}_{ij}+g_{k}+gl_{ik}+e_{ijk}$$ (1)

where *Y* is the DI of the *k*^*th*^ genotype tested in the *j*^*th*^ replication of the *i*^*th*^ environment, μ is the overall mean, *l* is the effect of the *i*^*th*^ environment, *b*(*l*) is the effect of the *j*^*th*^ replication nested in the *i*^*th*^ environment, *g* is the genetic effect of the *k*^*th*^ genotype, *gl* is the interaction effect of the *k*^*th*^ genotype and *i*^*th*^ environment, and *e* is the random experimental error. The comparison of the DI means among the different ILs was performed using the least significant difference (LSD) test [40] at the 5% level of significance.

### Genotyping for tracking the introgressed alien chromsomal segments

To track the presence of *H*. *praecox* chromosomal segments, genotyping was performed using the GBS method described by Elshire et al. [41] for the selected ILs and the parental lines, HA 89 and HA 458. All five highly heterozygous *H*. *praecox* accessions were excluded from the GBS experiment. Leaves were collected from four greenhouse-grown young plants of each selected sunflower lines, bulked, and freeze-dried. Genomic DNA was isolated from the freeze-dried tissues using the ‘DNeasy 96 plant kit’ (Qiagen, Valencia, CA, USA). DNA concentrations were measured using a NanoDrop 2000 Spectrophotometer (Thermo Fisher Scientific, Wilmington, DE, USA). DNA samples of each line (~2.0 μg) were sent to the Institute of Biotechnology, Cornell University, Ithaca, NY for GBS analysis. In brief, genomic DNA samples of individual ILs and recurrent parents were digested separately with *Eco*T22I, a restriction endonuclease that recognizes a six base-pair sequence (ATGCAT). The digested DNA fragments were then ligated to two types of adapters: a barcoded adapter to identify each sample and a common adapter with an *Eco*T22I compatible sticky end. The DNA samples were pooled and PCR was performed to amplify the ligated products using primers complementary to the ‘adapters’ sequences. The PCR products were then purified and loaded for sequencing on an Illumina Hiseq 2000 (Illumina, USA). Sequencing produced an average of 1,785,943 good barcode reads for the two recurrent parents and an average of 2,187,155 good barcode reads for the eight ILs. SNPs were extracted using the GBS discovery pipeline version 3.0.166 implemented in TASSEL software [42]. Briefly, tag counts were generated from Illumina sequencing fastq files using the ‘FastqTo-TagCountPlugin’. Tag counts were merged with ‘MergeMultipleTagCountPlugin’ (options: −c 3) and were aligned to the sunflower reference genome HA412.v1.0. (http://sunflowergenome.org) using the Burrow–Wheelers Alignment tool version 0.7.8-r455 [43] and converted into a ‘TagsOnPhysicalMap’ file for SNP calling using the TASSEL-GBS quantitative SNP caller. The GBS protocol identified 22,061 SNPs among the recurrent parents and the eight *H*. *praecox* ILs (S1 Table). The SNPs assigned to one of the 17 sunflower chromosomes were named with a prefix of S1 to S17, which corresponds to the respective chromosomes, followed by a number representing the physical position of the SNP on the genome. The SNPs that were unassigned to any of the 17 sunflower chromosomes, or had missing data in either of the parents, or showed polymorphism between HA 89 and HA 458 were removed, leaving a total of 10,530 SNP markers for further analysis.

### Phenotype and genotype tests for DM resistance

Phenotypic screening of the DM resistance was performed in the parents, HA 89 and HA 458, and in the selected H.pra 1 to H.pra 8 of *H*. *praecox* ILs using the North America (NA) *Plasmopara halstedii* race 734. This is a highly virulent race identified in USA in 2010 [44]. HA 458 is a known carrier of DM *R-*gene, *Pl*_*17*_. Resistance for DM in these lines was tested using the whole seedling immersion method in the greenhouse under control conditions [35,45]. The susceptible plants produced numerous white fungal spores on the abaxial surface of the cotyledons and true leaves, while the resistant plants lacked spores.

Genotyping of the parental lines, HA 89 and HA 458, and the eight selected ILs, H.pra 1 to H.pra 8 was performed using a simple sequence repeat (SSR) marker ORS963, and two single nucleotide polymorphism (SNP) markers, SFW04052 and SFW08268. These markers are tightly linked to the DM resistance gene *Pl*_*17*_ [35]. A polymerase chain reaction (PCR) for the SSR and SNP markers was performed as described by Qi et al. [46] and Qi et al. [35], respectively. The PCR reactions were run on a Peltier thermocycler (Bio-Rad Lab, Hercules, CA, USA) and the products were size segregated in an IR^2^ 4300/4200 DNA Analyzer with denaturing polyacrylamide gel electrophoresis (LI-COR, Lincoln, NE, USA).

## Results

### Hybridization and early generation selection for BSR resistance in the greenhouse

The F_1_ seed set varied among the five *H*. *praecox* accessions used in this study (Table 1). The highest number of F_1_ seeds was produced in the crosses with the two accessions of subspecies *hirtus* (13.8% each), followed by the two accessions of subspecies *runyonii* (3.9 and 6.6%), while the lowest number of seed set was observed in subspecies *praecox* (1.2%).

**Table 1:** F_1_ hybrid seed set from the crosses of NMS HA 89 with the selected basal stalk rot resistant plants from wild sunflower accessions of *H*. *praecox*.

Crosses	No. of florets pollinated	No. of seeds obtained	Seed set (%)
NMS HA89 × *H*. *praecox* subsp. *praecox* PI 413176	2131	26	1.2
NMS HA89 × *H*. *praecox* subsp. *runyonii* PI 435849	1602	63	3.9
NMS HA89 × *H*. *praecox* subsp. *runyonii* PI 468853	1679	111	6.6
NMS HA89 × *H*. *praecox* subsp. *hirtus* PI 468847	1383	191	13.8
NMS HA89 × *H*. *praecox* subsp. *hirtus* PI 435855	1721	238	13.8

Twenty-two to thirty-six F_1_ seeds derived from the crosses with wild *H*. *praecox* accessions were grown and tested for BSR resistance in the greenhouse (Table 2). The highest DI was observed in the susceptible check Cargill 270 (96%), which was followed by the recurrent parent HA 89 (36%). The F_1_ hybrid plants derived from the crosses with accessions PI 413176, PI 435849, and PI 435855 of the subspecies *praecox*, *runyonii* and *hirtus*, respectively, did not show any BSR symptoms. The F_1_ hybrid plants of the remaining two crosses with accession PI 468853 of subspecies *runyonii* and accession PI 468847 of subspecies *hirtus* had DI values of 22% and 25%, respectively, which were similar to the DI of the resistant checks HA 441 (DI 14%) and Croplan 305 (DI 18%).

**Table 2:** Sclerotinia basal stalk rot disease incidence in the recurrent parent, checks, and F_1_ plants derived from crosses with wild sunflower accessions of *H*. *praecox*.

Plant ID	Parents/checks/F_1_s	No. of plant tested	Disease incidence (%)
10–122	Cargill 270 (S-check)	48	96
10–001	HA 89 (recurrent parent)	38	36
10–121	HA 441 (R-check)	48	14
10–137	Croplan 305 (R-check)	44	18
10–136	(NMS HA89 × *H*. *praecox* subsp. *praecox* PI 413176)	22	0
10–132	(NMS HA89 × *H*. *praecox* subsp. *runyonii* PI 435849)	36	0
10–133	(NMS HA89 × *H*. *praecox* subsp. *runyonii* PI 468853)	36	22
10–134	(NMS HA89 × *H*. *praecox* subsp. *hirtus* PI 468847)	28	25
10–135	(NMS HA89 × *H*. *praecox* subsp. *hirtus* PI 435855)	36	0

The selected resistant F_1_ plants were used as the male parents to cross with HA 458 to obtain BC_1_ seeds. The screening of the BC_1_F_1_ plants resulted in only four BSR resistant plants derived from the crosses with accession PI 468853 of subspecies *runyonii*, and two from accession PI 468847 subspecies *hirtus*. These resistant BC_1_F_1_ plants were used as male parents in backcrosses to HA 89 to obtain BC_2_ seeds. The screening of the BC_2_F_1_ plants revealed that the progenies of the accession PI 468847 subspecies *hirtus* were susceptible to BSR. Seventy-one BC_2_F_1_ plants from the cross with accession PI 468853 of subspecies *runyonii* were screened for BSR resistance in the greenhouse, and finally 12 resistant plants were self-pollinated and advanced to the BC_2_F_2_ generation.

### Evaluation of BC_2_F_2_ populations for BSR resistance in the greenhouse

A total of eight BC_2_F_2_ populations derived from the crosses with *H*. *praecox* subsp. *runyonii* accession PI 468853 with enough seed set were evaluated for resistance to BSR during the winter of 2011 and early spring of 2012. Either 48 or 72 plants in each population were tested for BSR resistance with a total of 480 BC_2_F_2_ plants. Wide variation of DI was observed among the BC_2_F_2_ populations, ranging from 10.4 to 69.4%, with a mean DI of 45.7% across eight BC_2_F_2_ populations (Table 3).

**Table 3:** Summary of the Sclerotinia basal stalk rot tests of BC_2_F_2_ populations in the greenhouse derived from crosses with wild sunflower accessions of *H*. *praecox* subspecies *runyonii*.

Line/Plant ID	Pedigree	No. of plant tested	No. of dead plants	Disease incidence (%)
Cargill 270 (S-check)		12	10	83.3
HA 89 (recurrent parent)		12	10	83.3
HA 441 (R-check)		12	1	8.3
Croplan 305 (R-check)		12	1	8.3
11–291	HA89//HA458/(NMS HA89 × *H*. *praecox* subsp. *runyonii* PI 468853)	72	17	23.6
11–292	HA89//HA458/(NMS HA89 × *H*. *praecox* subsp. *runyonii* PI 468853)	72	34	47.2
11–293	HA89//HA458/(NMS HA89 × *H*. *praecox* subsp. *runyonii* PI 468853)	72	50	69.4
11–294	HA89//HA458/(NMS HA89 × *H*. *praecox* subsp. *runyonii* PI 468853)	72	27	37.5
11–295	HA89//HA458/(NMS HA89 × *H*. *praecox* subsp. *runyonii* PI 468853)	48	5	10.4
11–296	HA89//HA458/(NMS HA89 × *H*. *praecox* subsp. *runyonii* PI 468853)	48	28	58.3
11–297	HA89//HA458/(NMS HA89 × *H*. *praecox* subsp. *runyonii* PI 468853)	48	25	52.1
11–298	HA89//HA458/(NMS HA89 × *H*. *praecox* subsp. *runyonii* PI 468853)	48	32	66.7
Total of BC_2_F_2_		480	218	45.7

The DI scores of these eight populations were higher than the DI scores of 8.3% for both the resistant checks HA 441 and Croplan 305, suggesting segregation of BSR resistance in these early generation populations. A total of forty-one plants was selected from seven BC_2_F_2_ populations based on their BSR DI and advanced to the BC_2_F_3_ generation; nineteen were from 11–291, seven from 11–292, four each from 11–294 and 11–295, three each from 11–297 and 11–298, and a single plant from the 11–293 BC_2_F_2_ population (Table 3).

### Field evaluation of selected BSR resistant ILs

#### BC_2_F_3_ and BC_2_F_4_ evaluations

The selected 41 BC_2_F_3_ families along with the recurrent parent and checks were tested for BSR resistance in the field at Carrington, ND and Crookston, MN in 2012. An additional four BC_2_F_3_ families were also tested at Crookston in 2013. The two-year (2012 and 2013) mean DI of the susceptible check (Cargill 270), recurrent parent (HA 89), and resistant checks (HA 441, and Croplan 305) were 47.4, 33.0, 31.9, and 19.9%, respectively (S2 Table). Overall, 31 of 45 BC_2_F_3_ families had DI lower than both the resistant checks. Among the BC_2_F_3_ families, two had no infection, eighteen had a DI lower than 10%, and the remaining 11 families had a DI lower than 20%. A total of forty-four BSR resistant plants were selected from thirteen BC_2_F_3_ families, four plants each from 11-291-01, 11-291-05, 11-291-09, 11-291-33, 11-291-45, 11-291-65, 11-291-67, 11-294-21, and 11-295-01, three plants from 11-291-17, two plants each from 11-292-33 and 11-295-17, and a single plant from 11-291-57 BC_2_F_3_ family, and advanced to the BC_2_F_4_ generation.

The selected forty-four *H*. *praecox* BC_2_F_4_ plants were evaluated for BSR resistance at Crookston, MN in 2013. The mean DI scores for Cargill 270, HA 89, HA 441, and Croplan 305 were 72.6, 51.6, 28.6, and 34.9%, respectively (S3 Table). Forty out of 44 BC_2_F_4_ plants had lower DI scores than either of the resistant checks, HA 441 or Croplan 305. Among the BC_2_F_4_ families, 17 had ≤ 10% BSR DI, and three had no infection. A total of eight BSR resistant plants were selected and advanced to the BC_2_F_5_ generation.

#### BSR-resistant *H*. *praecox* ILs

**Fig 2:**
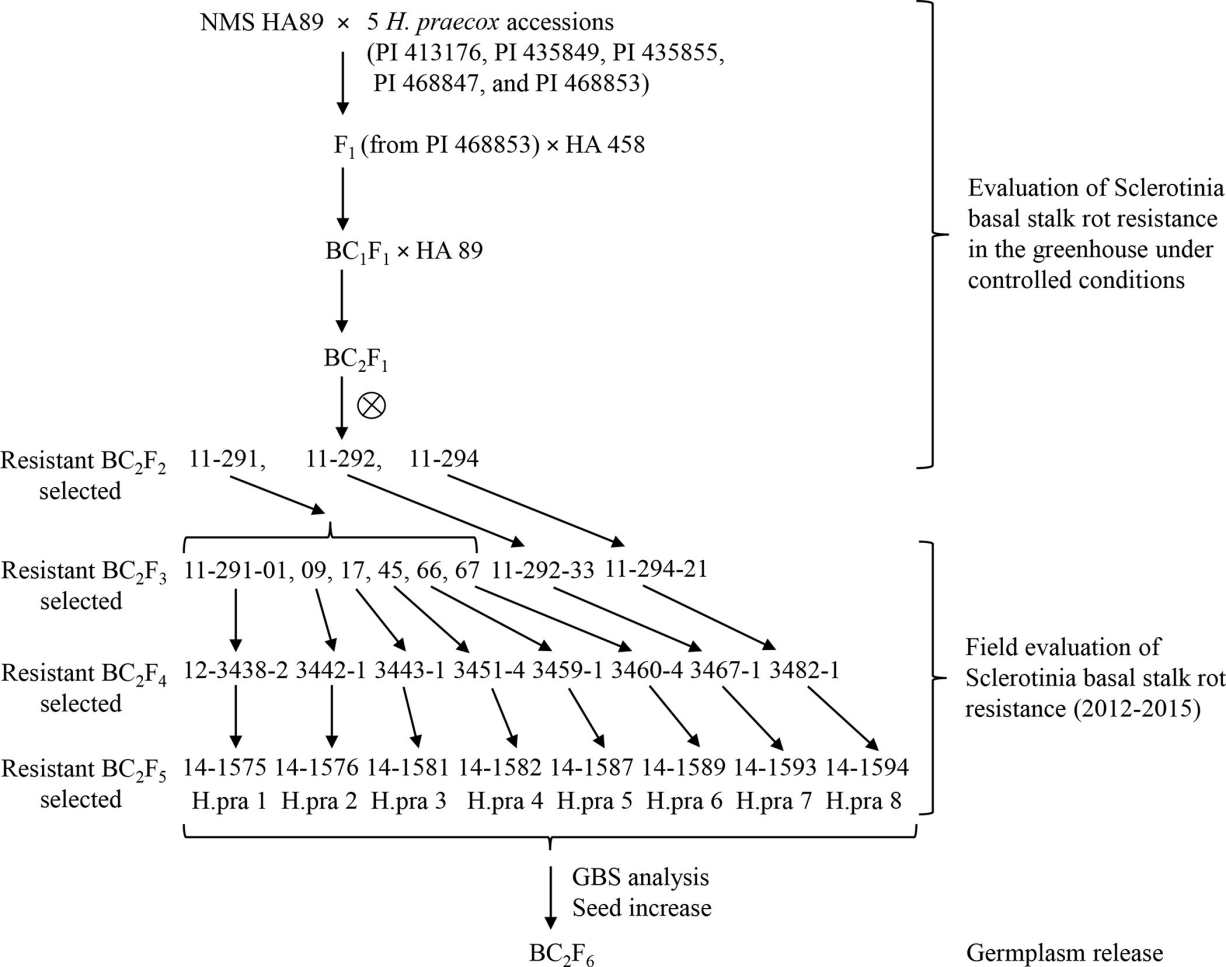
Schematic diagram showing the pedigree and selection of the eight Sclerotinia basal stock rot resistant sunflower introgression lines derived from the crosses of wild *H*. *praecox* species.

The eight selected *H*. *praecox* ILs, H.pra 1 to H.pra 8 (Fig 2) from the eight BC_2_F_4_ families, 12-3438-2, 12-3442-1, 12-3443-1, 12-3451-4, 12-3459-1, 12-3460-4, 12-3467-1, and 12-3482-1, were further evaluated for Sclerotinia BSR resistance in 2014 and 2015 at Carrington and Grandin, ND. The performance of these eight ILs and their ancestral families evaluated across seven environments (location and/or year) of North Dakota and Minnesota is summarized in Table 4.

**Table 4:** Sclerotinia basal stalk rot tests of selected introgression lines derived from crosses with wild sunflower species *H*. *praecox* at multiple locations of North Dakota and Minnesota from 2012 to 2015.

Line/Plant ID	Disease incidence (%)							
	Mean	2015 (BC_2_F_5_)		2014 (BC_2_F_4_)		2013 (BC_2_F_4_/F_3_)	2012 (BC_2_F_3_)	
		Grandin	Carrington	Grandin	Carrington	Crookston	Carrington	Crookston
Cargill 270 (S-check)	36.1	10.0	17.6	34.6	37.4	72.6	45.0	24.6
HA 89 (recurrent parent)	31.0	4.9	18.6	31.8	39.5	51.6	22.3	25.0
HA 441 (R-check)	19.5	2.1	3.8	29.7	6.8	28.6	39.2	27.8
Croplan 305 (R-check)	11.6	2.1	1.9	11.2	7.9	34.9	14.7	10.0
H.pra 1	7.8	11.5	1.9	9.5	9.5	3.1	13.3	0.0
H.pra 2	4.8	7.7	2.6	5.2	3.8	6.7	9.0	0.0
H.pra 3	11.1	38.5	10.4	10.6	2.0	4.2	0.0	4.0
H.pra 4	5.4	4.8	2.2	9.5	6.3	3.9	6.7	3.9
H.pra 5	1.2	0.0	0.0	1.4	0.0	0.0	8.3	0.0
H.pra 6	2.2	2.1	0.0	15.8	0.0	0.0	4.6	0.0
H.pra 7	2.2	6.7	0.0	NA	NA	3.3	0.0	0.0
H.pra 8	5.7	6.7	14.3	6.7	0.0	3.3	6.7	0.0
Mean±SE†	11.6±3.3	8.1±2.0	6.1±1.7	19.0±2.1	15.4±2.1	25.0±4.7	17.5±2.9	10.84±2.3
LSD (0.05)	8.8 ***	13.0 ***	- ns	17.5 ***	13.5 ***	17.0 ***	15.1 ***	13.7 ***

†SE: Standard error of means; NA: not available

***: significantly different at *p* < 0.001; ns: nonsignificant

Sclerotinia BSR prevalence varied greatly across the years and/or locations. The Crookston 2013 environment was the most conducive for BSR in sunflower with a mean DI of 25.0%, which was followed by Grandin 2014 and Carrington 2012 environments with mean DI scores of 19.0 and 17.5%, respectively. Overall, 2015 was less conducive for BSR disease development as manifested by the low mean DI scores of 6.1 and 8.1%, respectively, at the Carrington and Grandin locations (Table 4). The mean BSR DI of the eight *H*. *praecox* ILs ranged from 1.2 to 11.1%, while the scores were 36.1 and 31.0% for the susceptablie checks Cargill 270 and HA 89, and 19.5 and 11.6% for the resistant checks HA 441 and Croplan 305, respectively (Table 4). The mean BSR DI of the ILs was significantly lower than either one or both of the resistant checks, except the IL, H.pra 3 which had DI similar to the resistant check Croplan 305 (Table 4).

### Tracking *H*. *praecox* alien segments in the ILs

**Fig 3:**
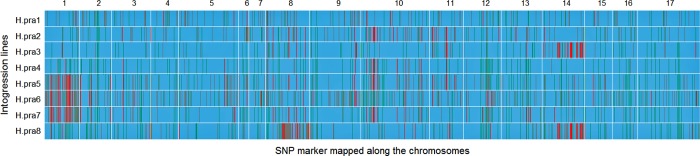
Graphical genotypes of the eight introgression lines showing the introgression regions on the 17 sunflower chromosomes. Blue colors represent the proportion of the cultivated sunflower genome. Red colors represent the *H*. *praecox* homozygous introgression regions, green colors are the heterozygous introgressions, and gray colors represent missing data.

Wide variation in the SNP distribution was observed throughout the sunflower genome of the ILs with the lowest in chromosome 6 (236 SNPs) and highest in chromosome 10 (1,034 SNPs) (Table 5). Out of 10,530 filtered SNPs, 806 were polymorphic between the recurrent parents and one or more of the ILs (S4 Table). Among the ILs H.pra 1, H.pra 2, H.pra 3, H.pra 4, H.pra 5, H.pra 6, H.pra 7, and H.pra 8, the number of polymorphic SNPs were 78, 176, 207, 113, 338, 255, 271 and 253, respectively (Table 5). Although, the number of polymorphic SNPs varied across the genomes of the ILs, a few common introgression regions were detected (Fig 3). Overall, the introduced *H*. *praecox* segments in the eight ILs were mainly recovered on chromosomes 1, 8, 10, 11, and 14 of the sunflower genome. Among the eight *H*. *praecox* ILs, the highest number of polymorphic SNPs was detected on chromosome 14 (133), followed by chromosome 1 (128), chromosome 8 (118), chromosome 10 (93), and chromosome 11 (50) of the sunflower genome (Table 6). Out of the 128 SNP markers recovered from the BSR-resistant donor parent on chromosome 1, 70 SNPs were shared among H.pra 5, H.pra 6, and H.pra 7 (57.4% of the polymorphic SNPs) (Table 6, S1 Fig). Most of these shared SNPs were distributed between the 13 to 150 Mb region on the physical map of chromosome 1, indicating common introgression regions on chromosome 1 (Table 6, S5 Table). In chromosome 8, a total of 118 SNP markers were recovered from the BSR resistant *H*. *praecox* parent, with the majority detected in the ILs H.pra 2, H.pra 3, H.pra 5, H.pra 7 and H.pra 8. A total of 32 SNPs were shared among H.pra 2, H.pra 3, H.pra 5, and H.pra 7, accounting for 69.6% of the total *H*. *praecox* alleles recovered on chromosome 8 in these ILs (Table 6, S1 Fig). Although 72 SNP markers were recovered on chromosome 8 in H.pra 8, only five were shared with the rest of the group, suggesting a unique introgression region in this IL (S5 Table).

**Table 5:** Tracking of the alien segments introduced from *H*. *praecox* in the highly basal stalk rot resistant germplasm lines using single nucleotide polymorphism markers developed using the genotyping-by-sequencing approach.

Line	Number of polymorphic SNP markers																	
	LG1 (594)*	LG2 (533)	LG3 (630)	LG4 (470)	LG5 (921)	LG6 (236)	LG7 (324)	LG8 (697)	LG9 (794)	LG10 (1034)	LG11 (558)	LG12 (608)	LG13 (652)	LG14 (675)	LG15 (474)	LG16 (445)	LG17 (885)	Total (10530)
H.pra 1	3	1	2	8	8	8	0	4	7	14	2	6	0	6	5	3	1	78
H.pra 2	0	0	3	0	1	8	0	39	10	68	28	5	5	5	1	2	1	176
H.pra 3	1	0	3	2	2	1	1	41	8	5	27	2	8	101	0	1	4	207
H.pra 4	4	6	2	0	7	0	0	7	8	70	2	5	0	0	0	2	0	113
H.pra 5	101	7	4	1	32	9	1	40	9	66	28	8	3	13	9	4	3	338
H.pra 6	89	7	6	1	29	13	1	9	30	14	10	13	2	14	9	4	4	255
H.pra 7	103	11	3	2	8	1	1	37	0	70	5	11	7	5	0	7	0	271
H.pra 8	7	0	14	2	4	1	1	72	4	8	23	3	2	102	2	4	4	253

*The number in parentheses are SNP markers detected by GBS

The intensity of the green color indicates the proportion of the polymorphism between the recurrent parent and the introgressed lines

**Table 6:** Distribution of the polymorphic SNP markers of *H*. *praecox* and the shared SNPs of the introgression lines in chromosomes 1, 8, 10, 11 and 14.

Chromosome		Total Polymorphic SNP	SNP distribution along physical regions (Mb)							Shared SNP	
Chr	Length (Mb)*		0–50	51–100	101–150	151–200	201–250	251–300	301–350	No. of SNP	Introgression lines
1	176.0	128	39	56	25	8				70	H.pra 5, 6, 7
8	192.1	118	15	14	51	38				32	H.pra 2, 3, 5, & 7
10	327.8	93	38	7	16	10	11	6	5	50	H.pra 2, 4, 5 & 7
11	208.7	50	23	17	6	4				7	H.pra 2, 3, 5 & 8
14	230.3	133	10	26	33	46	18			97	H.pra 3 & 8

* The physical length of chromosome taken from https://www.sunflowergenome.org/

Out of the 93 polymorphic SNPs on chromosome 10, 50 SNPs were shared among H.pra 2, H.pra 4, H.pra 5 and H.pra 7 (59.5% of the polymorphic SNPs), a common introgression region on chromosome 10 (Table 6, S1 Fig). Most of these shared SNPs were located between the 29 to 49 and 139 to 201 Mb regions on the physical map of chromosome 10 (Table 6, S5 Table). The highest number of *H*. *praecox* SNP markers were largely recovered on chromosome 14 (133 SNPs) in the H.pra 3 and H.pra 8 ILs (Table 6, Fig 3). A total of 97 SNPs were shared between the two ILs, H.pra 3 and H.pra 8, accounting for 91.5% of the total resistant donor alleles recovered on chromosome 14 in these ILs (Table 6, S1 Fig). Additional introgression regions were also observed in some of the *H*. *praecox* ILs on chromosome 11 (Table 6, Fig 3).

### DM resistance in the ILs

The sunflower inbred line, HA 458, used in the crossing scheme is resistant to DM disease conferred by the *Pl*_*17*_ gene, effective against all virulent *P*. *halstedii* races currently identified in the USA [35,47]. The eight ILs were genotyped using the three DNA markers, SFW04052, ORS963, and SFW08268 that are linked to the *Pl*_*17*_ gene. *Pl*_*17*_ was mapped to a 2.9-cM interval between SFW04052 and ORS963 [35]. SFW04052 was distal to *Pl*_*17*_ at 2.1 cM, while ORS963 was proximal to *Pl*_*17*_ at 0.8 cM in the genetic map. SFW08268 was downstream of ORS963 at 1.0 cM. Six of the eight ILs produced the same PCR pattern at three marker loci, while the remaining two lines, H.pra 4 and H.pra 6, had recombination events detected between SFW04052 and ORS963 (Table 7).

**Table 7:** Phenotypic disease response of downy mildew and marker tests of the introgression lines.

Line	DM score			DNA markers flanking *Pl*_*17*_		
	S	R	Phenotype	SFW04052	ORS963	SFW08268
HA 89	15	0	S	A	A	A
HA 458	0	16	R	B	B	B
H.pra 1	16	0	S	A	A	A
H.pra 2	0	20	R	B	B	B
H.pra 3	19	0	S	A	A	A
H.pra 4	0	25	R	**B**	**A**	A
H.pra 5	0	25	R	B	B	B
H.pra 6	0	25	R	**H**	**B**	B
H.pra 7	17	0	S	A	A	A
H.pra 8	20	0	S	A	A	A

S, susceptible; R, resistant; A, HA 89 PCR pattern; B, HA 458 PCR pattern; H, heterozygous. The *bold capital letters* indicate recombination between marker

Phenotypic evaluation of the ILs was conducted using isolate of the NA *P*. *halstedii* race 734, and the disease responses of the ILs were generally consistent with the marker data, except for H.pra 4 (Table 7). Lines H.pra 2 and H.pra 5 with all three DNA marker loci from HA 458 were homozygous resistant, and lines H.pra 1, H.pra 3, H.pra 7 and H.pra 8 with the three DNA marker loci from HA 89 were homozygous susceptible (Table 7). H.pra 6 was resistant to the disease although it was heterozygous at the SFW04052 marker locus. This could be the result of the HA 458 allele at ORS963 marker locus, which is the closest marker linked to *Pl*_*17*_ at a genetic distance of 0.8 cM. The phenotype of H.pra 4 was resistant, although it had the allele from HA 89 at the ORS963 locus. This result suggests the possibility that a crossover event occurred between the *Pl*_*17*_ gene and ORS963 marker.

## Discussion

In the present study, we used five highly BSR-resistant *H*. *praecox* accessions, one from subspecies *praecox* and two each from subspecies *runyonii* and *hirtus* to transfer BSR resistance into the cultivated sunflower. As predicted, the F_1_ hybrid seed set was very low for each cross (Table 1). In earlier studies, fewer than expected seed sets were reported in F_1_ interspecies hybrids between cultivated sunflower and the wild *H*. *praecox* subspecies due to the meiotic chromosomal aberrations [48,49]. Although our crossing program began with five highly BSR-resistant *H*. *praecox* accessions from three subspecies, we ended up with segregating progenies only from the cross involving PI 468853 *H*. *praecox* subsp. *runyonii*. Infertility of the segregating generations or reduced recombination between the chromosomes of the wild *H*. *praecox* subsp. *praecox* and *H*. *praecox* subsp. *hirtus* and cultivated sunflower might have eliminated progenies for BSR resistance evaluation.

BSR resistances have been successfully transferred from wild *Helianthus* species into cultivated sunflower background, and eight ILs have been developed from crosses of HA 89 with *H*. *praecox* through seven disease-screening cycles (F_1_ to BC_2_F_5_). A high disease pressure was used in the greenhouse screening trials in the early segregating generations (F_1_ to BC_2_F_2_) and only selected the highly resistant segregates to advance to the next generation. An intense selection pressure enhances the probability to recover the trait and favors the desired introgression fragment to be stable until the region becomes homozygous [50]. The ILs developed in this study largely showed stable BSR resistance across multi-location field screening trials in four years (Table 4). The mean DI in the eight lines was significantly lower than those of the susceptible check, Cargill 270, and the recurrent parent, HA 89. Most of the ILs were either significantly more resistant than one or both of the resistant checks, except the IL, H.pra3, which had a DI similar to the checks (Table 4). The prevalence of BSR disease varied across the field screening environments with the highest in 2013 and lowest in 2015, which became more evident from the BSR DI scores of the recurrent parent and both resistant and susceptible checks (Table 4). By contrast, with a few exceptions, the ILs consistently showed stable and superior resistance across environments, suggesting the successful transfer of novel Sclerotinia BSR resistance from wild *H*. *praecox* species. Nevertheless, variable level of BSR resistance has been observed among the eight ILs. BSR resistance in sunflower is controlled by quantitative genes with additive effects. When BSR resistance was transferred from wild species, the selected ILs might have integrated different partial resistance genes from wild species and resulted in different levels of resistance among selected ILs.

SNP variations are ubiquitous in the genome and are extremely suitable for a wide range of genomic studies [51]. GBS is an application of NGS technology that facilitates simultaneous discovery and genotyping of many SNP markers in crop genomes [41]. It is now routinely used for dissecting complex quantitative traits (for review Talukder et al. [9]; He et al. [52]) and, more recently, it has been demonstrated as a highly efficient tool for high-throughput tracking of introgressions [36,53–57]. In our study, the GBS analysis discovered a total of 10,530 filtered SNPs of which 806 unique SNPs were polymorphic between recurrent parents and one or more of the ILs. Because the selection of ILs was performed under intense BSR disease pressure, the retained alien segments in the cultivated sunflower background are likely associated with Sclerotinia resistance. The polymorphic SNPs were distributed across the entire genome of the ILs, which was expected for a polygenically controlled quantitative trait. However, the introduced *H*. *praecox* alien segments were mostly detected on chromosomes 1, 8, 10, 11 and 14 of the ILs in the cultivated sunflower background (Table 6, S5 Table). While there were common introgressions detected among a few lines by shared polymorphic SNPs (Table 6, S1 Fig), many introgressions were unique making each of the ILs a valuable resource for BSR resistance genes/quantitative trail loci (QTL). In an earlier study, Qi et al. [36] transferred Sclerotinia BSR resistance from two annual species, *H*. *argophyllus* and *H*. *petiolaris*, into cultivated sunflower and tracked alien introgressions using GBS-derived SNP markers. A comparative study revealed that out of 806 polymorphic unique SNP markers that detected alien segments of *H*. *praecox* in the current study, only 115 SNPs were common to the detected alien segments of either *H*. *argophyllus* or *H*. *petiolaris* in the previous study (S4 Table), suggesting transfer of novel Sclerotinia BSR resistance from wild *H*. *praecox* species.

Sclerotinia BSR resistance QTL have been previously mapped using candidate gene association mapping [10] and in biparental mapping populations derived from cultivated sunflower background [7–9]. Talukder et al. [10] reported a strong association of Sclerotinia BSR with orthologs of the *Arabidopsis thaliana COI1* (Coronatine Insensitive 1) gene, *HaCOI1*-*1* and *HaCOI1*-*2* located approximately at the positions 221.85 and 90.43 Mb regions, respectively, on the chromosome 14 of sunflower physical map, explaining 7.4% of phenotypic variation in the association mapping population. In our study, *H*. *praecox* alien segments were detected within ~2 kb of the *HaCOI1*-*1* gene in H.pra 3, H.pra 5 and H.pra 6, while alien segments were detected within ~2 kb near the *HaCOI1*-*2* gene in H.pra 3 and H.pra 8 ILs.

Talukder et al. [9] used GBS-derived SNP markers to map BSR resistance QTL in a sunflower recombinant inbred line (RIL) population developed from the cross of inbred lines HA 441/RHA 439. Two major QTL were identified on chromosomes 10 and 17 in multiple environments and each explained 31.6 and 20.2%, respectively, of the observed phenotypic variance in the RIL population. Our current study detected the *H*. *praecox* alien segment in H.pra 4, H.pra 5 and H.pra 7 within the tightly flanking SNP markers S10_281294015 and S10_288646223 (~7.35 Mb) of the BSR resistance QTL, *Qbsr-10*.*1* on chromosome 10. Overall, a significant number of wild *H*. *praecox* alien segments was detected along the entire genome of the selected ILs each possessing higher levels of Sclerotinia BSR resistance. Some of these introgressions were detected in regions of previously identified BSR resistance QTL; the majority of which were unique and might be associated with new BSR resistance. A detail QTL study will elucidate the role of these alien segments in the underlying genetic mechanism of BSR resistance in these lines. Efforts are underway to evaluate the mapping population developed from wild *H*. *praecox* species for BSR resistance in locations across North Dakota and Minnesota.

One of the cultivated sunflower parents used in the current study, HA 458, is resistant to downy mildew, another major sunflower disease of global importance. HA 458 possesses a DM resistant gene *Pl*_17_ that is highly effective against all known *P*. *halstedii* races thus far identified in the USA [34,35,47]. Although no additional effort was made to select DM resistance during IL development, H.pra 2, H.pra 4, H.pra 5, and H.pra 6 showed complete resistance to the highly virulent DM race 734 (Table 7). The selected ILs with dual resistance against two important sunflower diseases, Sclerotinia BSR and DM, represent a valuable genetic source for disease resistance breeding in sunflower.

Despite the high level of BSR resistance available in the wild *Helianthus* species, adequate utilization of this invaluable resource has been limited in sunflower breeding due to the linkage drag and different incompatibility barriers between cultivated and wild species. Gene introgression from secondary gene-pools coupled with high-throughput tracking of introgressions presented here will provide a unique opportunity to expand the genetic base of cultivated sunflower by exploiting genetic variability present in wild species, as well as ensuring a continuous supply of new sources of resistance feeding into breeding pipelines to maintain the sunflower as a viable major global oilseed crop.

## Supporting information

S1 Fig Vann diagrams showing the shared SNPs among *H. praecox* introgression lines in the different chromosomes. (TIF)

S1 Table Total SNP markers detected from genotype-by-sequencing protocol in recurrent parents and *H. praecox* sunflower intrgression lines genome. (XLSX)

S2 Table Summary of the Sclerotinia basal stalk rot tests of the BC2F3 families in inoculated field nurseries at Carrington and Crookston in 2012, and Crookston in 2013. (XLSX)

S3 Table Summary of the Sclerotinia basal stalk rot tests of the BC2F4 plants in inoculated field nurseries at Crookston in 2013. (XLSX)

S4 Table Unique polymorphic SNPs between recurrent parents and one or more of the H. praecox introgression lines. (XLSX)

S5 Table Polymorphic SNPs detected in chromosomes 1, 8, 10, 11, and 14 of the *H. praecox* sunflower intrgression lines genome. (XLSX)

## References

[1] Bolton MD, Thomma BP, Nelson BD. *Sclerotinia sclerotiorum* (Lib.) deBary: biology and molecular traits of a cosmopolitan pathogen. Mol Plant Pathol. 2006;7: 1–16. 10.1111/j.1364-3703.2005.00316.x 20507424

[2] Gulya TJ, Rashid K, Masirevic S. Sunflower diseases In: Schneiter AA, editor. Sunflower Technology and Production. Soil Science Society of America Inc, Madison, WI; 1997 pp. 263–379.

[3] Gulya TJ, Vick B, Nelson B. Sclerotinia head rot of sunflower in North Dakota: 1986 incidence, effect on yield and oil components, and sources of resistance. Plant Dis. 1989;73: 504–507.

[4] Talukder ZI, Hulke BS, Marek LF, Gulya TJ. Sources of resistance to sunflower diseases in a global collection of domesticated USDA plant introductions. Crop Sci. 2014;54: 694–705. 10.2135/cropsci2013.07.0506

[5] Bazzalo ME, Dimarco P, Martinez F, Daleo GR. Indicators of resistance of sunflower plant to basal rot (*Sclerotinia sclerotiomm*): Symptomatological, biochemical, anatomical, and morphological characters of the host. Euphytica. 1991;57: 195–205.

[6] Gulya TJ, Marek LF, Gavrilova V. Disease resistance in cultivated sunflower derived from public germplasm collections. In: Proceedings of the International Symposium "Sunflower Breeding on Resistance to Diseases, Krasnodar, Russia, June 23–24, 2010. The International Sunflower Association, Paris. pp 7–18.

[7] Davar R, Darvishzadeh R, Majd A, Ghosta Y, Sarrafi A. QTL mapping of partial resistance to basal stem rot in sunflower using recombinant inbred lines. Phytopathol Mediterr. 2010;49: 330–341.

[8] Amouzadeh M, Darvishzadeh R, Haddadi P, Aabdollahi Mandoulakani B, Rezaee Danesh Y. Genetic analysis of partial resistance to basal stem rot (*Sclerotinia sclerotiorum*) in sunflower. Genetika. 2013;45: 737–748. 10.2298/gensr1303737a

[9] Talukder ZI, Seiler GJ, Song Q, Ma G, Qi LL. SNP discovery and QTL mapping of Sclerotinia basal stalk rot resistance in sunflower using genotyping-by-sequencing. The Plant Genome. 2016; 9(3). 10.3835/plantgenome2016.03.0035 27902793

[10] Talukder ZI, Hulke BS, Qi LL, Scheffler BE, Pegadaraju V, McPhee K, et al Candidate gene association mapping of Sclerotinia stalk rot resistance in sunflower (*Helianthus annuus* L.) uncovers the importance of *COI1* homologs. Theor Appl Genet. 2014;127: 193–209. 10.1007/s00122-013-2210-x 24193356

[11] Cheres M, Knapp S. Ancestral origins and genetic diversity of cultivated sunflower: Coancestry analysis of public germplasm. Crop Sci. 1998;38: 1476–1482. 10.2135/cropsci1998.0011183X003800060012x

[12] Ladizinsky G. Founder effect in crop-plant evolution. Econ Bot. 1985;39:191–199. 10.1007/bf02907844

[13] Hahn V. Genetic variation for resistance to Sclerotinia head rot in sunflower inbred lines. Field Crops Res. 2002;77: 153–159. 10.1016/S0378-4290(02)00082-5

[14] Seiler GJ, Qi LL, Marek LF. Utilization of sunflower crop wild relatives for cultivated sunflower improvement. Crop Sci. 2017;57: 1–19. 10.2135/cropsci2016.10.0856

[15] Dußle CM, Hahn V, Knapp SJ, Bauer E. *Pl*_Arg_ from *Helianthus argophyllus* is unlinked to other known downy mildew resistance genes in sunflower. Theor Appl Genet. 2004;109: 1083–1086. 10.1007/s00122-004-1722-9 15221147

[16] Qi LL, Seiler GJ, Hulke BS, Vick BA, Gulya TJ. Genetics and mapping of the *R*_*11*_ gene conferring resistance to recently emerged rust races, tightly linked to male fertility restoration in sunflower (*Helianthus annuus* L.). Theor Appl Genet. 2012;125: 921–932. 10.1007/s00122-012-1883-x 22610307

[17] Qi LL, Foley ME, Cai XW, Gulya TJ. Genetics and mapping of a novel downy mildew resistance gene, *Pl*_*18*_ introgressed from wild *Helianthus argophyllus* into cultivated sunflower (*Helianthus annuus* L.). Theor Appl Genet. 2016;129: 741–52. 10.1007/s00122-015-2662-2 26747047

[18] Gong L, Hulke BS, Gulya TJ, Markell SG, Qi LL. Molecular tagging of a novel rust resistance gene *R*_*12*_ in sunflower (*Helianthus annuus* L.). Theor Appl Genet. 2013;126: 93–99. 10.1007/s00122-012-1962-z 22907633

[19] Zhang ZW, Ma GJ, Zhao J, Markell SG, Qi LL. Discovery and introgression of the wild sunflower-derived novel downy mildew resistance gene *Pl*_19_ in confection sunflower (*Helianthus annuus* L.). Theor Appl Genet. 2017;130: 29–39. 10.1007/s00122-016-2786-z 27677630

[20] Ma GJ, Markell SG, Song QJ, Qi LL. Genotyping-by-sequencing targeting of a novel downy mildew resistance gene *Pl*_*20*_ from wild *Helianthus argophyllus* for sunflower (*Helianthus annuus* L.). Theor Appl Genet. 2017;130: 1519–1529. 10.1007/s00122-017-2906-4 28432412

[21] Badouin H, Gouzy J, Grassa CJ, Murat F, Staton SE, Cottret L, et al The sunflower genome provides insights into oil metabolism, flowering and Asterid evolution. Nature. 2017;546: 148–152. 10.1038/nature22380 28538728

[22] Twyford AD, Ennos RA. Next-generation hybridization and introgression. Heredity. 2012;108: 179–189. 10.1038/hdy.2011.68 21897439 PMC3282392

[23] Schilling EE. *Helianthus* In: Flora of North America Editorial Committee (editors) Flora of North America North of Mexico. Oxford University Press, New York and Oxford 2006;21: 141–169.

[24] Turner BL. Taxonomy of the Texas sunflower (*Helianthus praecox*) Asteraceae. Phytologia. 2014;96: 107–109.

[25] Škorić D. FAO sunflower sub-network report 1984–1986 In: Škorić D, editor. Genetic evaluation and use of *Helianthus* wild species and their use in breeding programs. FAO, Rome, Italy 1987; pp. 1–17.

[26] Rönicke S, Hahn V, Horn R, Grone I, Brahn H, Friedt W. Interspecific hybrids of sunflower as sources of Sclerotinia resistance. Plant Breed. 2004;123: 152–157. 10.1046/j.1439-0523.2003.00925.x

[27] Block CC, Marek LF, Gulya TJ. Evaluation of wild Helianthus species for resistance to Sclerotinia stalk rot. In: Procedings of the 7th Annual Sclerotinia Initiative Meeting, Bloomington, MN. January 21–23, 2009. National Sclerotinia Initiative, Fargo, ND. Available from: http://www.ars.usda.gov/Research/docs.htm?docid=20392#Evaluation

[28] Block CC, Marek LF, Gulya TJ. Evaluation of wild Helianthus species for resistance to Sclerotinia stalk rot. In: Procedings of the 8th Annual Sclerotinia Initiative Meeting, Bloomington, MN. January 20–22, 2010. National Sclerotinia Initiative, Fargo, ND. Available from: http://www.ars.usda.gov/Research/docs.htm?docid$=$21051#evaluation

[29] Block CC, Gulya TJ, Marek LF. Identifying resistance to Sclerotinia stalk and root rot in perennial germplasm. In: Procedings of the APS Annual Meeting, Providence, RI. August 4–8, 2012. American Phytopathological Society Press, St. Paul, MN. p. 269. Available from: http://www.apsnet.org/meetings/Documents/2012_Meeting_Abstracts/aps12abP269.htm

[30] Marek LF, Block CC, Gardner CA. 2012 update: New sunflower genetic resources in the US national sunflower collection and potential use for crop improvement. In: Procedings of the 18th International Sunflower Conference, Mar del Plata, Argentina. February 27–March 1, 2012. The International Sunflower Association, Paris. pp. 1–6.

[31] Christov M. Contribution of interspecific and intergeneric hybridization to sunflower breeding. Helia. 2013;36: 1–18. 10.2298/hel1358001a

[32] Jan CC, Rutger JN. Mitomycin C- and streptomycin-induced male sterility in cultivated sunflower. Crop Sci. 1988;28: 792–795. 10.2135/cropsci1988.0011183X002800050014x

[33] Jan CC. Four sunflower nuclear male-sterile genetic stocks. Crop Sci. 1992;32: 1519–1519. 10.2135/cropsci1992.0011183X003200060061x

[34] Hulke BS, Miller JF, Gulya TJ, Vick BA. Registration of the oilseed sunflower genetic stocks HA 458, HA 459, and HA 460 possessing genes for resistance to downy mildew. J Plant Reg. 2010;4: 93–97. 10.3198/jpr2009.08.0426crgs

[35] Qi LL, Long YM, Jan CC, Ma GJ, Gulya TJ. *Pl*_*17*_ is a novel gene independent of known downy mildew resistance genes in the cultivated sunflower (*Helianthus annuus* L.). Theor Appl Genet. 2015;128: 757–767. 10.1007/s00122-015-2470-8 25673143

[36] Qi LL, Long Y, Talukder ZI, Seiler GJ, Block CC, Gulya TJ. Genotyping by sequencing uncovers the introgression alien segments associated with Sclerotinia basal stalk rot resistance from wild species—I. *Helianthus argophyllus* and *H*. *petiolaris*. Front Genet. 2016;7: 219 10.3389/fgene.2016.00219 28083014 PMC5183654

[37] Gulya TJ, Radi S, Balbyshev N. Large scale field evaluations for Sclerotinia stalk rot resistance in cultivated sunflower. In: Velasco L, editor. Procedings of the 17th International Sunflower Conference, Córdoba, Spain. June 8–12, 2008. The International Sunflower Association, Paris. pp. 175–179.

[38] Schneiter AA, Miller JF. Description of sunflower growth stages. Crop Sci. 1981;21: 901–903.

[39] SAS Institute. The SAS System for Windows. Version 9.4. SAS Institute Inc, 2013 Cary, NC.

[40] Steel RGD, Torrie JH. Principles and procedures of statistics, 2nd edition, 1980 McGraw Hill, New York.

[41] Elshire RJ, Glaubitz JC, Sun Q, Poland JA, Kawamoto K, Buckler ES, Mitchell SE. A robust, simple genotyping-by-sequencing (GBS) approach for high diversity species. PLoS One. 2011;6(5): e19379 10.1371/journal.pone.0019379 21573248 PMC3087801

[42] Bradbury PJ, Zhang Z, Kroon DE, Casstevens TM, Ramdoss Y, Buckler ES. TASSEL: Software for association mapping of complex traits in diverse samples. Bioinformatics. 2007;23: 2633–2635. 10.1093/bioinformatics/btm308 17586829

[43] Li H, Durbin R. Fast and accurate short read alignment with Burrows–Wheeler transform. Bioinformatics. 2009;25: 1754–1760. 10.1093/bioinformatics/btp324 19451168 PMC2705234

[44] Gulya TJ, Markell S, McMullen M, Harveson B, Osborne L. New virulent races of downy mildew: distribution, status of DM resistant hybrids, and USDA sources of resistance. In: Proceedings of the 33rd Sunflower Research Forum, Fargo ND. January 12–13, 2011. National Sunflower Association, Mandan, ND. Available from: http://www.sunflowernsa.com/uploads/resources/575/gulya_virulentracesdownymildew.pdf

[45] Gulya TJ, Miller JF, Viranyi F, Sackston WE. Proposed internationally standardized methods for race identification of *Plasmopara halstedii*. Helia. 1991;14: 11–20.

[46] Qi LL, Gulya TJ, Seiler GJ, Hulke BS, Vick BA. Identification of resistance to new virulent races of rust in sunflower and validation of DNA markers in the gene pool. Phytopathol. 2011;101: 241–249. 10.1094/PHYTO-06-10-0162 20879847

[47] Gilley MA, Markell SG, Gulya TJ, Misar CG. Prevalence and virulence of Plasmopara halstedii (downy mildew) in sunflowers. In: Proceedings of the 38th Sunflower Research Forum, Fargo, ND. January 12–13, 2016. National Sunflower Association, Mandan, ND. Available from: http://www.sunflowernsa.com/uploads/research/1277/Prevalence.Downey_Gilley.etal_2016.rev.pdf

[48] Atlagić J, Terzić S. Cytogenetic study of an F1 sunflower interspecific hybrid (Helianthus annuus x Helianthus praecox). In: Velasco L, editor. Proceedings of the 17th International Sunflower Conference, Córdoba, Spain. June 8–12, 2008. The International Sunflower Association, Paris. pp. 721–724.

[49] Yushkina LL, Nesterova EV, Kirichenko VV, Dolgova TA, Popov VN. Cytogenetic analysis of interspecific hybrid *Helianthus praecox* × *H*. *annuus*, its parental forms, and two backcrosses. Cytol Genet. 2009;43: 33–37. 10.3103/S009545270901006X 19663314

[50] Breton C, Serieys H, Bervillé A. Gene transfer from wild *Helianthus* to sunflower: topicalities and limits. OCL. 2010;27: 104–114. 10.1051/ocl.2010.0296

[51] Rafalski A. Applications of single nucleotide polymorphisms in crop genetics. Curr Opin Plant Biol. 2002;5: 94–100. 10.1016/S1369-5266(02)00240-6 11856602

[52] He J, Zhao X, Laroche A, Lu ZX, Liu H, Li Z. Genotyping-by-sequencing (GBS), an ultimate marker assisted selection (MAS) tool to accelerate plant breeding. Front Plant Sci. 2014;5: 484 10.3389/fpls.2014.00484 25324846 PMC4179701

[53] Tiwari VK, Wang S, Sehgal S, Vrána J, Friebe B, Kubaláková M, et al SNP discovery for mapping alien introgressions in wheat. BMC Genomics. 2014;15: 273 10.1186/1471-2164-15-273 24716476 PMC4051138

[54] Wendler N, Mascher M, Noh C, Himmelbach A, Scholz U, Ruge-Wehling B, et al Unlocking the secondary gene-pool of barley with next-generation sequencing. Plant Biotechnol J. 2014;12: 1122–1131. 10.1111/pbi.12219 25040223

[55] Arbelaez JD, Moreno LT, Singh N, Tung CW, Maron LG, Ospina Y, et al Development and GBS-genotyping of introgression lines (ILs) using two wild species of rice, *O*. *meridionalis* and *O*. *rufipogon*, in a common recurrent parent, *O*. *sativa* cv. Curinga. Mol Breed. 2015;35: 81 10.1007/s11032-015-0276-7 25705117 PMC4328105

[56] Winfield MO, Allen AM, Burridge AJ, Barker GLA, Benbow HR, Wilkinson PA, et al High-density SNP genotyping array for hexaploid wheat and its secondary and tertiary gene pool. Plant Biotechnol J. 2016;14: 1195–1206. 10.1111/pbi.12485 26466852 PMC4950041

[57] King J, Grewal S, Yang C, Hubbart S, Scholefield D, Ashling S, et al A step change in the transfer of interspecific variation into wheat from *Amblyopyrum muticum*. Plant Biotechnol J. 2017;15: 217–226. 10.1111/pbi.12606 27459228 PMC5258861

