# Supplementary figures and images for: Introgression and monitoring of wild Helianthus praecox alien segments associated with Sclerotinia basal stalk rot resistance in sunflower using genotyping-by-sequencing
Source: PLoS One. 2019 Mar 1;14(3):e0213065. doi: 10.1371/journal.pone.0213065 (PMC6396933; doi:10.1371/journal.pone.0213065)

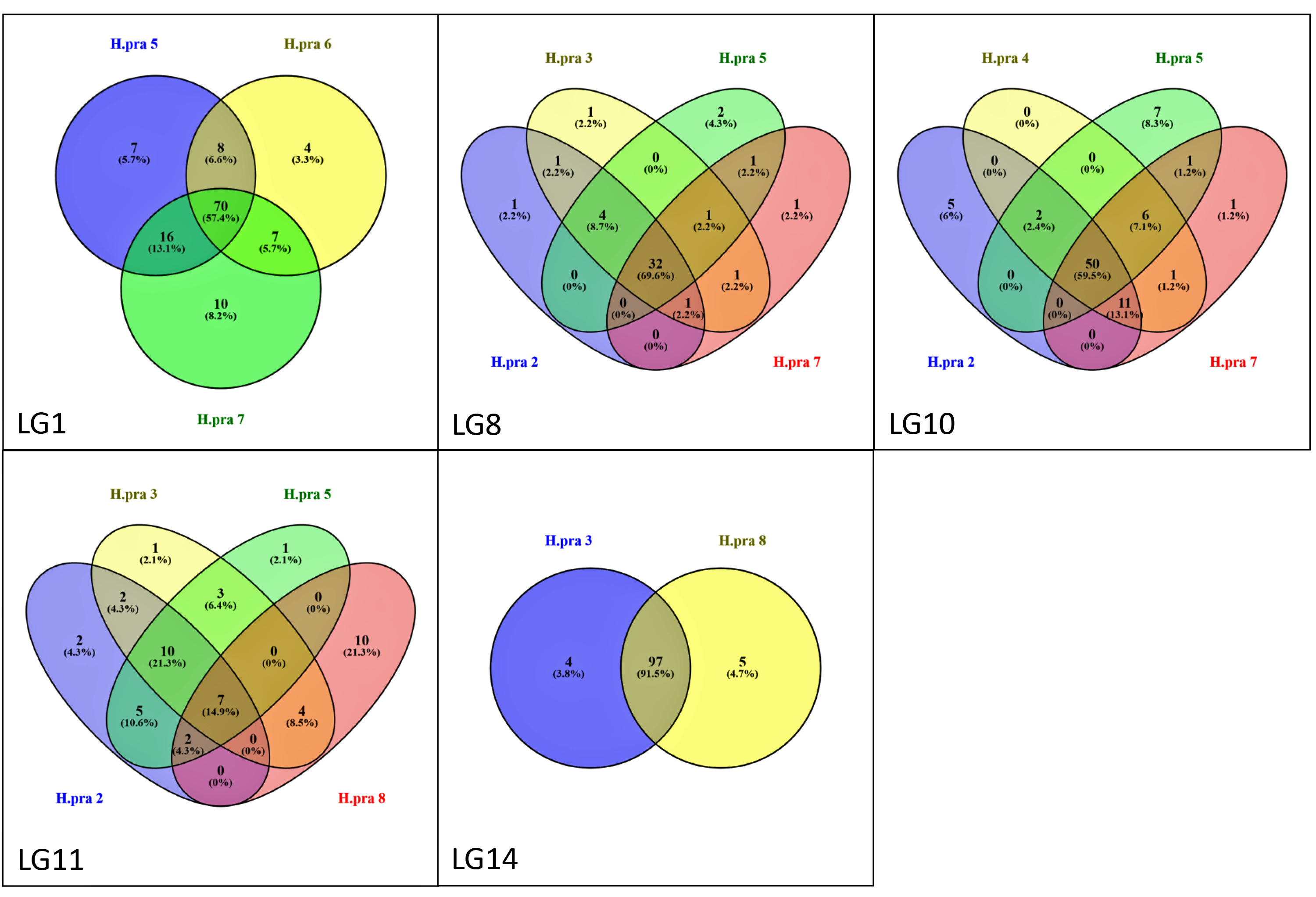

Supplement: S1 Fig — (TIF) [file pone.0213065.s001.tif]
